# Translation, cross-cultural adaptation, and validation of the sino-nasal outcome test (snot)-22 for Finnish patients

**DOI:** 10.1007/s00405-020-06297-w

**Published:** 2020-08-20

**Authors:** Anni Koskinen, Sari Hammarén-Malmi, Jyri Myller, Marjukka Mäkelä, Elina Penttilä, Timo Pessi, Tuomo Puhakka, Antti Raappana, Rami Taulu, Sanna Toppila-Salmi, Paula Virkkula, Maija Hytönen

**Affiliations:** 1 Department of Otorhinolaryngology- Head and Neck Surgery, Helsinki University Hospital and University of Helsinki, PO Box 263, 00029 Helsinki, Finland; 2 Department of Otorhinolaryngology, Päijät-Häme Central Hospital, Lahti, Finland; 3 Section of General Practice, Department of Public Health, Copenhagen University, Copenhagen, Denmark; 4 Department of Otorhinolaryngology, Head and Neck Surgery, Kuopio University Hospital and University of Eastern Finland, Kuopio, Finland; 5 Elisa Oyj, Healthcare Solutions, Helsinki, Finland; 6 Department of Otorhinolaryngology, Head and Neck Surgery, Turku University Hospital and University of Turku, Turku, Finland; 7 Department of Otorhinolaryngology and Head and Neck Surgery, Oulu University Hospital, Oulu, Finland; 8 PEDEGO Research Unit, University of Oulu, Oulu, Finland; 9 Department of Otorhinolaryngology, Head and Neck Surgery, Tampere University Hospital and University of Tampere, Tampere, Finland; 10 Skin and Allergy Hospital, University of Helsinki and Helsinki University Hospital, Helsinki, Finland

**Keywords:** Sino-nasal outcome test, SNOT-22, Validation, Translation, HRQoL

## Abstract

**Purpose:**

The Sino-Nasal Outcome Test-22 (SNOT-22) is the most commonly used disease-specific quality of life questionnaire in rhinology. The purpose of this prospective study was to translate and validate SNOT-22 into Finnish.

**Methods:**

The validation process followed the guidelines proposed for cross-cultural adaptation of health-related measures of quality of life. The study consisted of three groups: rhinologic out-patients (*N* = 96), FESS patients (*N* = 49) and healthy controls (*N* = 79). Out-patient and FESS groups completed the questionnaire twice (answers A and B), out-patients after two weeks and FESS patients after 3 months. Validity, reliability and responsiveness were evaluated.

**Results:**

The mean SNOT-22 sum score of the out-patient questionnaires were 35.3 points (answer A) and 32.4 points (answer B). ICC in out-patient group was 0.879. For the FESS patients, the mean pre- and postoperative (answer A and B) SNOT-22 sum scores were 46.8 and 21.9 points, respectively (*p* < 0.0001). The mean SNOT-22 of healthy controls was 8.9 points. The out-patients (answer A) and healthy controls had statistically significant difference in SNOT-22 scores (*p* < 0.0001).

**Conclusions:**

The results of our study show that the validated Finnish version of the SNOT-22 questionnaire demonstrates good validity, reliability and responsiveness.

## Introduction

Chronic rhinosinusitis (CRS) is a common medical problem affecting approximately 5–15% of people in Europe and United States [1–3]. Physicians from primary care to a wide range of specialists encounter these patients in their everyday work. CRS has a severe impact on the quality of life (QoL), which is comparable with other chronic diseases, such as asthma, chronic obstructive pulmonary disease (COPD) and diabetes [2].

Treatment of CRS is primarily conservative and surgery is warranted for patients refractory to medical therapy. Treatment effectiveness is evaluated utilizing sinonasal imaging and nasal endoscopy as well as different patient-reported outcome measures (PROMs) most commonly assessing the disease-specific health-related QoL (HRQoL). The use of HRQoL questionnaires is rapidly growing in studies of clinical effectiveness and quality of care, but also in clinical practise. A valid measure of rhinosinusitis patients’ health status and quality of life is required for the complete assessment of treatment effectiveness as well as a comparison of results of different studies.

In rhinology the most commonly used HRQoL questionnaire is Sino-Nasal Outcome Test-22 (SNOT-22). There exist several other HRQoL tools but SNOT-22 has been shown to be the most suitable due to its reliability, validity, responsiveness and ease of use [4]. Although originally designed for use in CRS it has also shown to be suitable as a measure of outcome after septal surgery [5].

The current SNOT-22 was developed in 2009 from modification of SNOT-20, by National Comparative Audit of Surgery for Nasal Polyposis and Rhinosinusitis Royal College of Surgeons of England [6]. SNOT-20 again is a modified version of the 31-Item Rhinosinusitis Outcome Measure (RSOM-31), originally developed in the University of Washington [7].

Considering the unique nature and features of different cultures and patients, it is essential to be able to use a HRQoL questionnaire translated and validated for the particular language. To be successful, the validation process requires a systematic approach to the translation and cross-cultural adaptation. To date SNOT-22 has been translated and validated in several languages e.g., Spanish, Russia, Arabic, Italian and Danish [8–12].

The aim of this study was to translate and validate the SNOT-22 to be used with Finnish-speaking patients. We assessed the reliability, validity and responsiveness of the translated SNOT-22 questionnaire.

## Materials and methods

SNOT-22 is composed of CRS-related items, which evaluate the severity of complaints that the patient has been experiencing over the past two weeks. All items are scored on a scale from 0 to 5, 0 meaning patient is not bothered by the symptom at all, 5 meaning the symptom is as worse as it can be. The sum of each item results in a minimum score of 0 and a maximum score of 110. The higher the score the worse are the symptoms and vice versa. The questions can be divided into two main categories: questions about physical symptoms (12 questions) which cover rhinologic symptoms as well as ear and facial symptom, and questions about general health and quality of life (10 questions) which cover sleep function and psychological issues.

The research team consisted of Finnish researchers from Finland’s all five University Hospitals and one Central Hospital, Skin and Allergy Hospital and the Finnish Institute for Health and Welfare. The study was carried out between February and October 2016. The results have been reported in Finnish in a Finnish medical journal Duodecim [13]. Validation and translation process were performed according to the guidelines proposed for cross-cultural adaptation of health-related measures of quality of life [14, 15]. Translation process consisted of translation, back-translation, review by a translation and retranslation committee and pre-testing.

In our study one professional translator translated the questionnaire from English into Finnish and another professional translator back-translated it into English. Despite this qualified process, the study group found the Finnish version of the questionnaire peculiar. Therefore, one of the study members translated the original questionnaire from English into Finnish, which was back-translated into English by a professional translator. The study group evaluated that the meaning of the words was preserved. Again, the Finnish version was back-translated into English by a translator who was both a native Finnish and English speaker. Finally, the study group compared this version to the final Finnish version and noticed that even though different words had been used, the meaning of the words had not changed. This Finnish version was pre-tested with five rhinologic out-patient patients, where each patient autonomously filled out this version of the SNOT-22 and discussed the wording and meaning of each item with the senior clinician.

After the translation process, we performed a prospective study to validate the Finnish version of SNOT-22. The study was approved by the Ethics Committee at the Helsinki University Hospital (§241/9.12.2015, DNRO 396/13/03/02/15). Each patient gave written informed consent to participate in the study. The study composed of three groups.

*Group I* consisted of adult (18 years or older) rhinologic out-patients who had one of the following diagnosis: chronic rhinosinusitis, nasal polyposis, septal deviation or chronic rhinitis. Finnish language skill of each patient was evaluated by the recruiting physician. The inclusion criterion was that the patients had to be able to understand and complete the questionnaire. If applicable, patients were informed of the study and completed the SNOT-22 questionnaire (answer A). Patients received a second SNOT-22 questionnaire to be completed two weeks after the first questionnaire (answer B). They also received another questionnaire with a transition rating scale assessing sinonasal symptoms and general health as a whole. Patients were instructed to compare their symptoms and general health to their status two weeks earlier. The options in this questionnaire were 1 = much better, 2 = a little better, 3 = about the same, 4 = a little worse and 5 = much worse than two weeks ago. Patients were instructed to send these second questionnaires in a return envelope by mail to the recruiting unit. To maximize the response rate the recruiting physician, send each patient a reminder text message two weeks after completing the first questionnaire.

*Group II* consisted of adult (18 years or older) patients undergoing functional endoscopic sinus surgery (FESS) due to chronic or recurrent rhinosinusitis and/or nasal polyposis. Patients underwent either endoscopic opening of the maxillary sinus or ethmoidectomy. Other inclusion criteria were the same as in group I. The first SNOT-22 questionnaire was conducted on the day of the surgery (answer A). Upon discharge, the patients got the second SNOT-22 questionnaire and the questionnaire with a transition rating scale, assessing sinonasal symptoms and general health as a whole, to be completed three months after the surgery (answer B) and a return mail envelope. In the questionnaire with the transitioning scale, the patients were instructed to compare their symptoms and general health to their preoperative status (scale from 1–5, as in Group I). Again, to maximize the response rate the recruiting physician, send each patient a reminder text message three months after completing the first questionnaire.

*Group III* consisted of adult controls (18 years or older) recruited from study hospitals’ personnel or close circle of the research team members. In addition to complete the SNOT-22 questionnaire, they were asked four questions with yes or no answer. These questions were: Whether (1) the subject has a chronic rhinologic disorder deduced by himself or diagnosed by a medical professional, (2) the subject had undergone any rhinologic surgery, (3) the subject had suffered from upper respiratory tract infection during the past two weeks (e.g., flu, sinus or ear infection) or symptoms of allergic rhinitis, and (4) the subject had been using any medication to rhinologic disorder or symptoms during the past 2 weeks. The 2 weeks interval was chosen being equivalent with SNOT-22.

### Statistical analysis

The statistical analyses were performed by a professional statistician. To assess the validity, the ability of the SNOT-22 to reflect differences between known groups, Mann–Whitney *U* test was used. The reliability of the SNOT-22 was evaluated by assessing internal consistency and test–retest reproducibility. Internal consistency refers to the way individual items relate to each other, whereas test–retest reproducibility measures the stability of an instrument over time with repeated testing. Cronbach's alpha reliability coefficient was used to represent and evaluate the internal consistency. The minimum acceptable score is 0.7 [16]. The interclass correlation coefficient (ICC) for intra-rater reliability [17] was used to evaluate the test–retest reliability in the out-patient group (group I) among patients whose symptom status had remained unchanged (transitioning scale 3) between the two questionnaires (answer A and B).

Responsiveness of the SNOT-22 score was evaluated using the Wilcoxon’s Signed-Rank by comparing the change in the SNOT-22 score between A and B answers for the FESS patients.

In cases where answers to single questions were missing, the mean value of given answers was calculated and the missing values replaced by this [6]. If at least half of the answers were missing, the patient was excluded from the analysis. If the patient had answered two or more values to one question, the mean of these values was calculated and used in the analysis (Hopkins, C. Personal Communication of unpublished data, 2016).

## Results

The group I consisted of 110 rhinologic out-patients on a routine visit. A- and B answers were obtained from 96/110 (87.3%) patients, 52 women and 44 men. The mean age of the patients was 49.8 (range 18.3–82.5) years. The mean SNOT-22 score was 35.3 (range 2–75.4) on the out-patient visit (answer A). The mean SNOT-22 score two weeks later (answer B) was 32.4 (range 1–75).

The group II consisted of 75 FESS-patients. A- and B-answers were obtained from 49/75 (65.3%) patients, 30 women and 19 men. The mean age of the patients was 47.2 (range 18.6–79.3) years. Pre-operatively the mean SNOT-22 score (answer A) was 46.8 (range 12–94); men 43.8 (range 16–87) and women 50.5 (range 12–94). Three months post-operatively the mean SNOT-22 score (answer B) was 21.9 (range 1–80.7); men 21.8 (range 1–80.7) and women 22.4 (range 3–80.7). The difference between pre- and postoperative SNOT-22 scores was statistically significant (*p* < 0.0001).

In group III altogether 139 people were recruited. Of these 50 (50/139, 36.0%) reported of having a chronic rhinologic disorder deduced by himself or diagnosed by a medical professional and/or having undergone previous rhinologic surgery and/or having used any medication to rhinologic disorder and/or symptoms during the past two weeks. These 50 were excluded from the study. Of the included 89 controls, 67 were women and 22 men, and the mean age was 40.4 (range 21–63) years. The mean SNOT-22 score was 10.4 (range 0–43). Ten (10/89, 11.2%) of the controls reported that they had had upper respiratory tract infection and/or allergic rhinitis during the last two weeks. The mean SNOT-22—score of this subgroup (called flu/all—group) was 22.0 (range 6–43).

The subgroup healthy-controls included 79 controls, 60 women and 19 men, who had answered’no’ to all four specified questions. The mean SNOT-22—score of this group was 8.9 (range 0–37); in men and women 6.6 (range 0–28) and 9.6 (range 0–37), respectively.

**Fig. 1:**
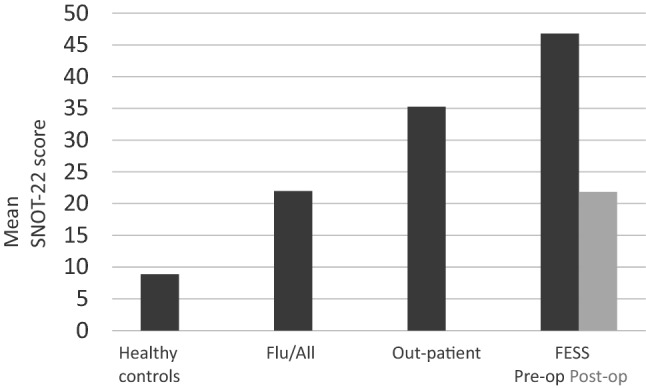
The mean SNOT-22—scores of the healthy-controls, flu/all (allergy)—group, out-patients—group and FESS—group. In the out-patient group the score is from the first questionnaire (answer A)

**Fig. 2:**
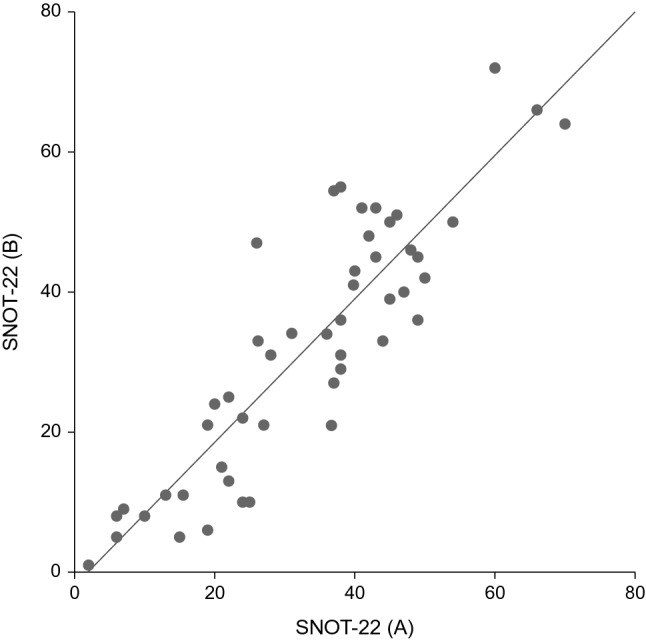
The mean SNOT-22—scores of the out-patients (answers A and B) whose symptom status had remained unchanged

The Figs. 1, 2 shows the mean SNOT-22—scores of the healthy-controls, flu/all—group, out-patients—group and FESS—group.

Validity was assessed by comparing the mean value of the total SNOT-22 scores of answer A in the outpatient group (group I) (35.3) with the healthy-control subgroup (group III) (8.9). There was a statistically significant difference between these groups (*p* < 0.0001).

Cronbach’s alpha was used to evaluate the internal consistency of the Finnish version of the SNOT-22. In the out-patients- and FESS-patients—groups in the A-answers the values were 0.897–0.930, and in B-answers 0.941–0.953, respectively. The Cronbach’s alpha in the control group was 0.895.

ICC was used to evaluate the test–retest reliability. Number of patients in the outpatient group (group I) who had answered transitioning scale 3, meaning their symptom status had not changed, was 49. ICC in this group was 0.879, representing good reliability [18].

None of the patients had chosen the same numerical value for all the questions but there was a variation of the chosen numbers. None of the respondents reported that they had had difficulties in understanding the questions.

## Discussion

Both in research and in clinical practise there is a growing interest to use validated HRQoL questionnaires when assessing clinical effectiveness and quality of care. In rhinology SNOT-22 is one of the most frequently used HRQOL instruments due to its reliability and usability [4]. As it combines both sinonasal‐specific and general health questions it helps us to evaluate, not just the disease severity but also the effect of symptoms on the patient’s daily life. SNOT-22 use is recommended especially when assessing which patients would benefit from surgery and again when assessing the outcome of surgery [4]. To date, SNOT-22 has been translated and validated in several countries [6, 8–12, 19, 20].

Our aim was to translate, validate and culturally adapt the SNOT-22 into the Finnish language to enable its use by otorhinolaryngologists both in research and clinical practise in Finland. We followed the guidelines proposed for cross-cultural adaptation of health-related measures of quality of life [14, 15].

The Finnish SNOT-22 questionnaire was capable in differentiating patients with the sinonasal disease and healthy controls and in this way demonstrating a good validity. The mean values of the scores of patients with the sinonasal disease were significantly different from those of healthy individuals. The out-patients’ mean SNOT-22 score was 35.3. The mean SNOT-22 score among healthy controls in our study was 8.9–10.4 among all controls, including the ones reporting flu or allergy symptoms during the past two weeks. In other studies, the mean scores of controls vary from 4.5 in Spain to 16.8 in Lithuania [8, 20]. A study assessing SNOT-22 in a control population, with 250 non‐CRS adults, reported a mean SNOT-22 value of 12.0, the mean values of women exceeding those of men [21]. Even though our healthy controls had lower mean values, we had the same gender trend; women having higher mean values than men, 9.6 and 6.6, respectively.

Two types of reliability were assessed in our study, internal consistency and test–retest reproducibility. We found good internal consistency scores (Cronbach alpha > 0.8) of all answers in each group. ICC, reflecting the test–retest reproducibility, was 0.879. Thus, a strong correlation was obtained between the scores of the initial test and the retest examination. These results are in accordance with the results presented by others [11, 20, 21].

According to the results of our study, the Finnish version of the SNOT-22 questionnaire is also capable of measuring changes in patients’ HRQoL after surgical intervention. The mean pre-operative score (46.8) was statistically significantly higher than the score 3 months post-operatively (21.9) (*p* < 0.0001), which demonstrates the responsiveness of the instrument. The mean change was 24.9. The corresponding values, pre-op-post-op = change, in Lithuanian, English and Russian validation studies were 52.4- 22.5 = 29.9, 41.7- 25.5 = 16.2, 67.6- 18.1 = 49.5, respectively. Comparing the numbers alone is not reasonable, due to putatively different extent of both disease and surgery performed. However, in all these studies the SNOT-22 was able to detect clinical changes over time [11, 20, 21].

### Limitations

As a CRS assessment tool SNOT-22 has few shortcomings itself. Being relatively long, it takes time and demands concentration from the patients to complete it. In our study, most patients completed the questionnaires either in the waiting room or at home so the external influence was reduced to a minimum.

SNOT-22 also includes questions about sleep function and psychological issues which similarly relate to numerous other confounding conditions such as sleep apnea or depression. These questions might confuse some patients about whether they should attempt to differentiate between rhinosinusitis and other conditions as the cause of their symptoms. A weakness of this study is the lack of data on patients’ comorbidities such as the ones mentioned above. However, no patient-reported difficulties in answering questions involving general health.

Finally, SNOT-22 does not seem to correlate well with objective measures of disease severity and, therefore, should not be used alone in decision making but together with CT and/or endoscopy findings [22, 23].

## Conclusion

SNOT-22 questionnaire combined with imaging and endoscopy findings is an effective tool to assess rhinologic patients’ disease burden and outcome of a given treatment. In Finland, there has not existed a validated questionnaire to evaluate the quality of life in patients with chronic rhinosinusitis, until now. Our validation process produced a Finnish version of SNOT-22 which has adequate validity, reliability and responsiveness.

## Data Availability

The datasets used and/or analysed during the current study are available from the corresponding author on reasonable request.

## References

[1] Pleis JR, Lethbridge-Cejku M (2007). Summary health statistics for U.S. adults: National health interview survey, 2006. Vital Health Stat 10.

[2] Hastan D, Fokkens WJ, Bachert C (2011). Chronic rhinosinusitis in europe–an underestimated disease. A GA(2)LEN study. Allergy.

[3] Bhattacharyya N (2011). Incremental health care utilization and expenditures for chronic rhinosinusitis in the united states. Ann Otol Rhinol Laryngol.

[4] Morley AD, Sharp HR (2006). A review of sinonasal outcome scoring systems: which is best?. Clin Otolaryngol.

[5] Buckland JR, Thomas S, Harries PG (2003). Can the sino-nasal outcome test (SNOT-22) be used as a reliable outcome measure for successful septal surgery?. Clin Otolaryngol Allied Sci.

[6] Hopkins C, Gillett S, Slack R (2009). Psychometric validity of the 22-item sinonasal outcome test. Clin Otolaryngol.

[7] Piccirillo JF, Merritt MG, Richards ML (2002). Psychometric and clinimetric validity of the 20-item sino-nasal outcome test (SNOT-20). Otolaryngol Head Neck Surg.

[8] de los Santos G, Reyes P, del Castillo R (2015). Cross-cultural adaptation and validation of the sino-nasal outcome test (SNOT-22) for spanish-speaking patients. Eur Arch Otorhinolaryngol.

[9] Asiri M, Alokby G (2019). Validation and cross-cultural adaptation of the sinonasal outcome test (SNOT)-22 for the arabian patient population. Cureus.

[10] Mozzanica F, Preti A, Gera R (2017). Cross-cultural adaptation and validation of the SNOT-22 into italian. Eur Arch Otorhinolaryngol.

[11] Eisenbach N, Matot S, Nemet A, Sela E, Marshak T, Ronen O (2020). Sino-nasal outcome test-22: cross-cultural adaptation and validation in Russian speaking patients. Clin Otolaryngol.

[12] Lange B, Thilsing T, Al-kalemji A (2011). The sino-nasal outcome test 22 validated for danish patients. Dan Med Bull.

[13] Hytönen M, Hammaren-Malmi S, Myller J (2017). Tautikohtaisen elämänlaatumittarin validointi- esimerkkinä nenä- ja sivuontelotautikohtainen SNOT-22-mittari. Duodecim.

[14] Guillemin F, Bombardier C, Beaton D (1993). Cross-cultural adaptation of health-related quality of life measures: literature review and proposed guidelines. J Clin Epidemiol.

[15] Wild D, Grove A, Martin M (2005). Principles of good practice for the translation and cultural adaptation process for patient-reported outcomes (PRO) measures: Report of the ISPOR task force for translation and cultural adaptation. Value Health.

[16] Aaronson N, Alonso J, Burnam A (2002). Assessing health status and quality-of-life instruments: attributes and review criteria. Qual Life Res.

[17] Gwet K (2008) Intrarater reliability. In: D'Agostino RB, Sullivan L, Massaro J (eds) Wiley encyclopedia of clinical trials 10.1002/9780471462422.eoct631

[18] Koo TK, Li MY (2016). A guideline of selecting and reporting intraclass correlation coefficients for reliability research. J Chiropr Med.

[19] Caminha GP, Melo Junior JT, Hopkins C (2012). SNOT-22: Psychometric properties and cross-cultural adaptation into the portuguese language spoken in brazil. Braz J Otorhinolaryngol.

[20] Vaitkus S, Padervinskis E, Balsevicius T (2013). Translation, cross-cultural adaptation, and validation of the sino-nasal outcome test (SNOT)-22 for lithuanian patients. Eur Arch Otorhinolaryngol.

[21] Erskine SE, Hopkins C, Clark A (2017). SNOT-22 in a control population. Clin Otolaryngol.

[22] Ryan WR, Ramachandra T, Hwang PH (2011). Correlations between symptoms, nasal endoscopy, and in-office computed tomography in post-surgical chronic rhinosinusitis patients. Laryngoscope.

[23] Wabnitz DA, Nair S, Wormald PJ (2005). Correlation between preoperative symptom scores, quality-of-life questionnaires, and staging with computed tomography in patients with chronic rhinosinusitis. Am J Rhinol.

